# Cardiac Tamponade in the Setting of a Thymoma

**DOI:** 10.7759/cureus.4952

**Published:** 2019-06-20

**Authors:** Abdul Khan, Akriti G Jain, Mohammed FaisalUddin, Neelam Khetpal, Jason D'Souza

**Affiliations:** 1 Internal Medicine, Florida Hospital, Orlando, USA; 2 Internal Medicine, Deccan College of Medical Sciences, Hyderabad, IND; 3 Cardiology, University of Missouri Kansas City/St. Luke's Health System, Kansas City, USA

**Keywords:** thymoma, pericardial effusion, hemorrhagic pericardial effusion, mediastinal mass, cardiac tamponade

## Abstract

Thymoma is the most common neoplasm originating in the anterior mediastinum and accounts for a quarter of all mediastinal tumors. A pericardial effusion is an uncommon initial manifestation present in approximately 20% of patients. However, our patient had hemorrhagic pericardial effusions causing a cardiac tamponade with bilateral pleural effusions, nodular left pleural based masses, and ascites. In this report, we describe the unique features of our case and its management.

## Introduction

Thymomas are the most common neoplasms originating in the anterior mediastinum and account for a quarter of all mediastinal tumors. The clinical presentation of a thymoma varies from being an asymptomatic accidental finding, to manifestations of paraneoplastic syndromes and symptoms caused by thoracic spread of the tumor. Pleural and pericardial effusions can occur in disseminated cases; however, a spontaneous hemorrhagic pericardial effusion complicated by cardiac tamponade is a very rare finding [1]. In this report, we describe the case of a thymoma with extensive pleural invasion and large pericardial effusions causing cardiac tamponade.

## Case presentation

**Figure 1:**
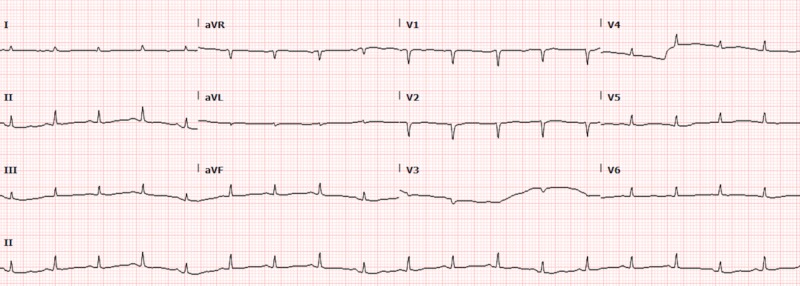
Electrocardiogram (EKG) showing electrical alternans and low voltage complexes

**Figure 2:**
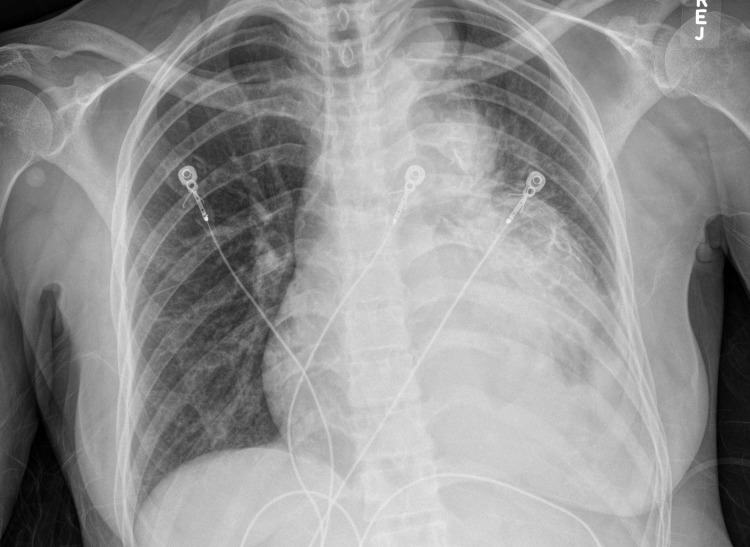
Chest X-ray showing cardiomegaly and left sided pleural effusion

**Figure 3:**
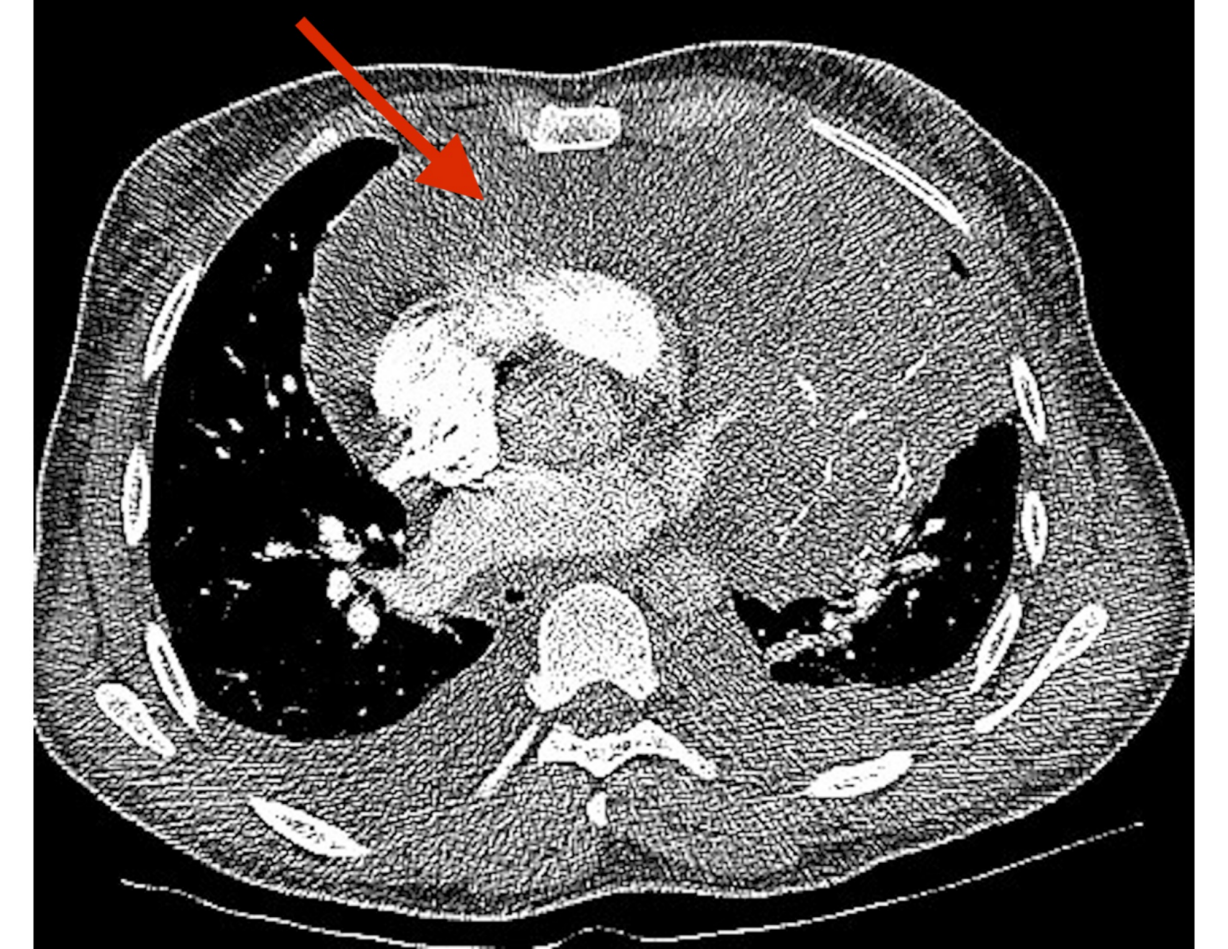
Computed tomography (CT) of the chest showing anterior mediastinal mass (arrow)

**Figure 4:**
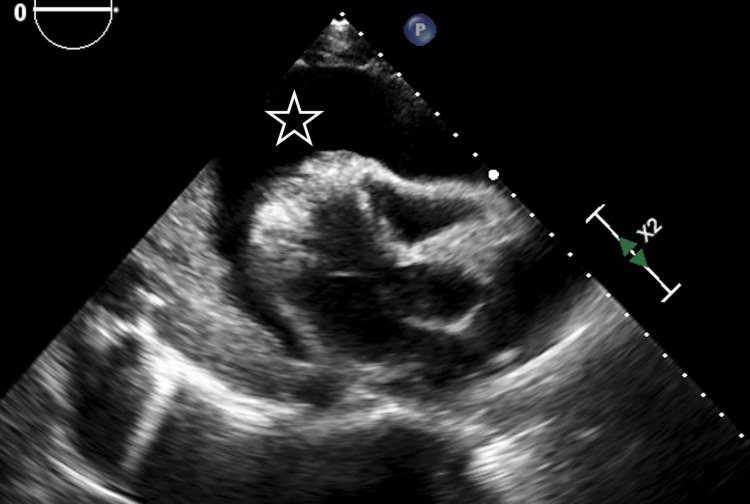
Transthoracic echocardiography (TTE) showing pericardial effusion (star)

**Figure 5:**
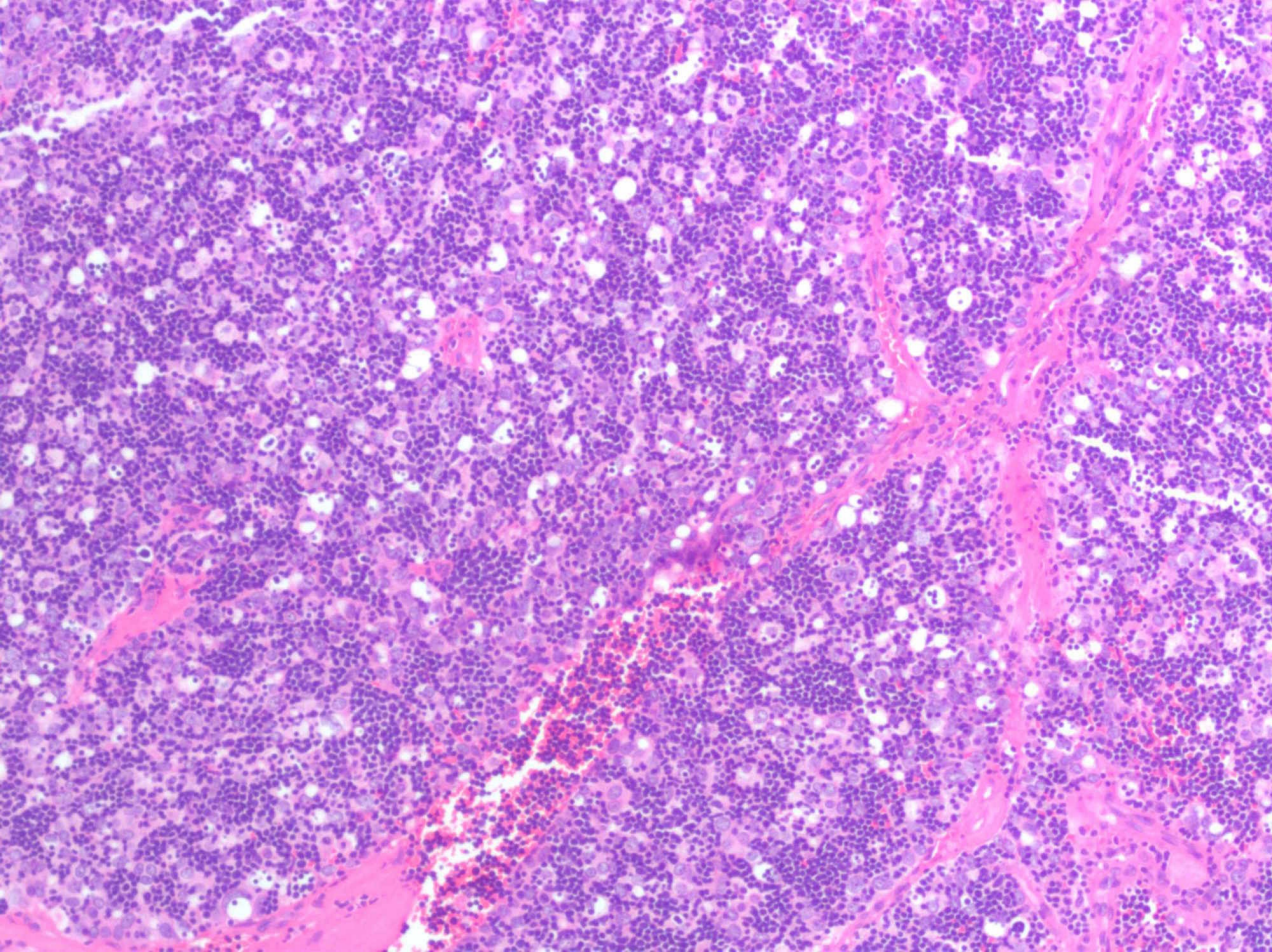
Histologic appearance of the resected tumor showing sheets of epithelial cells

A 47-year-old woman with a past medical history of asthma presented to the emergency room with fatigue, bilateral leg swelling and abdominal distention for two weeks. On physical examination, she was hypotensive, tachycardic with a heart rate of 119 beats per minute, had distended veins over her forehead with jugular vein distension (12 cm), muffled heart sounds, and pulsus paradoxus. Laboratory evaluation revealed mildly elevated erythrocyte sedimentation rate (ESR), C-reactive protein (CRP) and transaminitis. Her electrocardiogram (EKG) showed sinus tachycardia, electrical alternans and low voltage QRS complexes as noted in Figure 1. A chest X-ray was suggestive of cardiomegaly with left-sided pleural effusion, as seen in Figure 2, and a subsequent computed tomography (CT) scan of the chest revealed a large anterior mediastinal mass with extensive pleural invasion and bronchovascular encasement involving the pulmonary vessels (Figure 3). The CT scan was also remarkable for a large caliber pericardial effusion. Due to the patient’s hemodynamic instability with findings of a pericardial effusion, an emergent transthoracic echocardiogram (TTE) was performed which was suggestive of cardiac tamponade with diastolic collapse of the right ventricle (Figure 4). Cardio-thoracic surgery was consulted, and an emergent pericardial window was performed with drainage of 1500 ml of bloody pericardial fluid. Intraoperatively, tumor deposits were found on the pleura, and specimens were collected for pathologic analysis which showed sheets of epithelial cells in the background of numerous lymphocytes. The immunohistochemistry patterns of the epithelial cells were consistent with an invasive thymoma, type B2 (Figure 5).

The patient had tolerated the procedure well and had remarkable symptomatic improvement. Following the diagnosis of a thymoma, she underwent a positron emission tomography (PET) scan which showed extensive hypermetabolism in the anterior mediastinum and the left pleural cavity. The patient’s tumor was deemed surgically unresectable due to the extensive bronchial and pulmonary venous encasement on the left side. In view of her extensive disease and inaccessibility of surgical resection, a decision was made to initiate induction chemotherapy with cyclophosphamide, doxorubicin and cisplatin (CAP regimen) followed by radiotherapy or surgery depending on the response. The patient, however, had opted for holistic treatment options and chose to decline the initiation of chemotherapy. She is being followed with serial surveillance CT scans of the chest, and her disease is showing signs of expansion over the last year.

## Discussion

Thymomas account for approximately 20%-25% of all mediastinal tumors [2]. The overall incidence of thymomas is rare with only 0.15 cases per 100,000 being identified [3]. One-third to one-half of the patients with thymoma are asymptomatic, and 33% of patients present with local symptoms related to the involvement of surrounding structures like chest pain, chest discomfort, dyspnea, and superior vena cava syndrome [4]. Pericardial effusion is an uncommon initial manifestation, present in approximately 20% of patients. However, our patient had hemorrhagic pericardial effusion causing cardiac tamponade with bilateral pleural effusions, nodular left pleural-based masses, and ascites. On review of literature, only seven similar cases have previously been reported.

The Masaoka staging system is a widely applied tool in studies of thymoma, given the tumor rarity and histopathologic heterogeneity. It has been utilized in major studies which provide data supporting treatment decisions. Based on Masaoka staging, the thymoma in our case is a stage III tumor with macroscopic invasion into the pericardium and pleura [5]. The histological classification of thymic epithelial tumors identifies the tumor in our patient as the World Health Organisation (WHO) type B2 thymoma, with more frequent clustering of epithelial cells [6]. In January 2018, the American Joint Committee on Cancer, established the staging of thymomas based on the tumor, node, and metastasis (TNM) system to provide a common platform for comparison of data from different series. The TNM stage for our case was stage IV B (T2, N2 M1a) [7]. The stage of a thymoma and its complete resectability are factors that influence its prognosis. The prognosis for stage III (Masaoka staging) tumors appears to be grim with high rates of recurrence following resection [8].

In our case, the patient not only presented with the typical symptoms of dyspnea and cough, but also with unusual findings of bilateral lower extremity edema and abdominal distention indicating right and left sided heart failure. The tumor expansion caused pericardial effusion with subsequent cardiac tamponade, causing symptoms of heart failure and obstructive shock. Our case report highlights the importance of considering a thymoma as a differential cause of cardiac tamponade, besides idiopathic, drug-induced, tuberculosis-related, or radiation-induced pericarditis causing massive pericardial effusions [9-10]. An emergent drainage of the pericardial fluid was warranted in our patient due to the impending hemodynamic compromise. The diagnosis of malignant pericardial effusion was made by means of pericardiocentesis. There has only been limited experience of echocardiography in cases of thymic tumors metastatic to the pericardium, with rare reports of a pericardial hyperechogenic mass being seen [1,11]. Unlike some benign primary cardiac neoplasms, there are no echogenic features specific enough to diagnose a metastatic tumor invading the pericardium.

The possibility of complete surgical resection of a thymoma is determined by the extent of tumor invasion or adhesions to surrounding structures. Review of the literature shows that complete resection of a thymoma had a statistically significant impact on five-year survival [12]. Complete surgical resection is the initial treatment decision for all cases of a thymoma if preoperative evaluation suggests resectability and low risk of complications. For stage I and stage II (TNM staging) thymomas, tumor resection followed by post-operative radiotherapy for high-risk tumors is the preferred treatment option. Stage IIIA thymomas should be treated with surgery either initially or after neoadjuvant therapy. The treatment of stage IIIB (TNM staging) thymomas may include a combination of chemotherapy, radiation and/or surgery. In cases of unresectable thymomas with extensive pleural and pericardial spread and encasement of bronchovascular structures as in our case, systemic treatment options like radiotherapy and chemoradiotherapy have shown to improve survival. Thymomas are usually extremely radiosensitive, and chemoradiotherapy is the available option for invasive metastatic disease or inoperable tumors [13]. The tumors can be revaluated for surgical resection after initial chemoradiotherapy. Despite complete resection, thymomas have a tendency for late recurrence. For recurrent disease, surgery, radiation, and/or chemoradiation can be considered.

The options for chemotherapy include cyclophosphamide, doxorubicin, and cisplatin (CAP) or cisplatin and etoposide (PE). In the future, immunotherapy promises to be a valuable treatment option for thymic tumors as indicated in the Phase II study analyzing response to pembrolizumab [14]. 

## Conclusions

A thymoma with extensive pleural spread and pericardial effusions causing cardiac tamponade, as in our case, is an extremely rare presentation with insufficient data regarding the preferred treatment approach. The prognosis of such cases is poor with high rates of recurrence. Further information from similar case reports will help contribute to data regarding the presentation and clinical behavior of these tumors.
